# Mesenchymal Stem Cells Attenuate Diabetic Lung Fibrosis via Adjusting Sirt3-Mediated Stress Responses in Rats

**DOI:** 10.1155/2020/8076105

**Published:** 2020-02-04

**Authors:** Yang Chen, Fuping Zhang, Di Wang, Lan Li, Haibo Si, Chengshi Wang, Jingping Liu, Younan Chen, Jingqiu Cheng, Yanrong Lu

**Affiliations:** ^1^Key Laboratory of Transplant Engineering and Immunology, West China Hospital, Regenerative Medicine Research Center, Sichuan University, Chengdu 610041, China; ^2^Research Core Facility, West China Hospital, Sichuan University, Chengdu 610041, China; ^3^Department of Orthopaedics, West China Hospital, Sichuan University, Chengdu 610041, China

## Abstract

Diabetes affects a variety of organs such as the kidneys, eyes, and liver, and there is increasing evidence that the lung is also one of the target organs of diabetes and imbalance of Sirt3-mediated stress responses such as inflammation, oxidative stress, apoptosis, autophagy, and ER stress may contribute to diabetic lung fibrosis. Although previous studies have reported that mesenchymal stem cells (MSCs) have beneficial effects on various diabetic complications, the effect and mechanisms of MSCs on diabetes-induced lung injury are not clear. In this study, the STZ-induced diabetes model was constructed in rats, and the effect and potential mechanisms of bone marrow MSCs on diabetic lung fibrosis were investigated. The results revealed that fibrotic changes in the lung were successfully induced in the diabetic rats, while MSCs significantly inhibited or even reversed the changes. Specifically, MSCs upregulated the expression levels of Sirt3 and SOD2 and then activated the Nrf2/ARE signaling pathway, thereby controlling MDA, GSH content, and iNOS and NADPH oxidase subunit p22^phox^ expression levels in the lung tissue. Meanwhile, high levels of Sirt3 and SOD2 induced by MSCs reduced the expression levels of IL-1*β*, TNF-*α*, ICAM-1, and MMP9 by suppressing the NF-*κ*B/HMGB1/NLRP3/caspase-1 signaling pathway, as well as regulating the expression levels of cleaved caspasese-3, Bax, and Bcl2 by upregulating the expression level of P-Akt, thereby inhibiting the apoptosis of the lung tissue. In addition, MSCs also regulated the expression levels of LC3, P62, BiP, Chop, and PERK, thereby enhancing autophagy and attenuating endoplasmic reticulum stress. Taken together, our results suggest that MSCs effectively attenuate diabetic lung fibrosis via adjusting Sirt3-mediated responses, including inflammation, oxidative stress, apoptosis, autophagy, and endoplasmic reticulum stress, providing a theoretical foundation for further exploration of MSC-based diabetic therapeutics.

## 1. Introduction

Diabetes mellitus (DM) is a chronic metabolic disease characterized by persistent blood hyperglycemia; it causes profound long-term effects on a variety of organs, such as the kidneys, heart, brain, eyes, and liver [1–5]. In recent years, increasing evidence suggests that the lung is also one of the target organs for diabetic microangiopathy with DM [6–11]. With the steady increase in the incidence of diabetes in an aging population, more and more pulmonary dysfunction is likely to be attributed to diabetic pulmonary complications [12], but few studies have addressed the role of diabetic lung injury and its therapeutics.

It has been recognized that diabetes can induce various pathological responses, one of which is the development of fibrosis [13]. Tissue fibrosis initially results from tissue injury caused by pathological stimuli and is followed by the predominant extracellular matrix (ECM) accumulation [14]. It is currently believed that the tissue fibrosis caused by hyperglycemia involves disorders of multiple pathways, such as oxidative stress, NF-*κ*B pathway, TGF-*β* pathway, autophagy, apoptosis, and endoplasmic reticulum (ER) stress [15–22]. Sirtuin 3 (Sirt3) is a member of NAD^+^-dependent deacetylase; it is a key regulator of the mitochondrial respiratory chain and plays an important role in the pathophysiology of various diseases, such as diabetes and metabolic syndrome, and aging [23]. Existing studies have indicated that overexpression of Sirt3 is able to inhibit fibrosis in a variety of animal disease models [24–26].

In diabetes pathogenesis, Sirt3 plays a protective role and involves a variety of stress responses. For example, Sirt3 could ameliorate oxidative stress and mitochondrial dysfunction after intracerebral hemorrhage in diabetic rats [27], alter the NF-*κ*B-dependent inflammatory pathway in the proteinuric kidney disease [28], and prevent lung fibrosis by inhibiting alveolar epithelial cell mitochondrial DNA damage and apoptosis [29]. Recent studies have also identified that Sirt3 plays multifaceted roles in the regulation of autophagy [30] and protects pancreatic *β*-cells from ER stress-induced apoptosis and dysfunction [31]. Thus, Sirt3 may serve as an important target for inhibiting the tissue fibrosis induced by diabetes via regulating various stress responses such as inflammation, oxidative stress, apoptosis, autophagy, and ER stress.

MSCs harbor differentiation potential, immunosuppressive properties, and anti-inflammatory effects and are considered an ideal candidate cell type for the treatment of DM [32]. MSCs are found to ameliorate various diabetic complications, such as diabetic cardiomyopathy, retinopathy, nephropathy, peripheral neuropathy, and foot ulcer [33–37]. In our previous works, we also found that MSCs ameliorate hyperglycemia-induced endothelial injury [38, 39] and diabetic kidney injury [40]. However, there are few papers that have reported the effects and potential mechanisms of MSCs on Sirt3 and diabetic lung injury. Therefore, the present study is aimed at investigating the protective effect and possible mechanisms of MSCs against diabetic lung injury in rats.

## 2. Materials and Methods

### 2.1. Ethical Approval

Male Sprague-Dawley rats were purchased from Chengdu Dossy Experimental Animal Co. Ltd. (Chengdu, China). All experiments involving animal subjects were performed in accordance with guidelines approved by the Animal Care and Use Committee of West China Hospital, Sichuan University.

### 2.2. Isolation of Bone Marrow MSCs

To generate bone marrow MSCs, bone marrow mononuclear cells were harvested by flushing the tibiae and femurs of 3-week-old male Sprague-Dawley rats (50–55 g body weight) with phosphate-buffered solution (PBS). Bone marrow MSCs were cultured in complete medium (90% Dulbecco's modified Eagle's medium-low glucose+10% fetal bovine serum) (HyClone, Logan, USA) and grown with 5% CO_2_ in a humidified atmosphere at 37°C, identified as described previously [39]. Bone marrow MSCs were passaged every 2 to 3 days and were used for transplantation at passage three or four.

### 2.3. Experimental Design

Eight-week-old male Sprague-Dawley rats were used in this study. Before the experiments, the rats were housed for 1 week to adapt to the experimental animal facility with an ambient temperature of 22–25°C and were allowed access to water and food. A total of 18 rats, of which 12 were grouped for diabetes induction and 6 animals were grouped as controls, were used. The diabetic rat model was induced by a single intraperitoneal injection of STZ (Sigma, St. Louis, USA) dissolved in the sodium citrate buffer at 55 mg/kg body weight into rats after overnight fasting. Control rats received an equal volume of citric acid buffer. One week after the injection, rats with fasting blood glucose contents exceeding 16.7 mM were deemed diabetic. Six weeks after the induction, the diabetic rats were randomly divided into diabetes mellitus+phosphate-buffered saline (DM+PBS) and diabetes mellitus+bone marrow MSC (DM+BMSC) groups (*n* = 6 in each group). For the rats in the DM+BMSC group, 5 × 10^6^ MSCs were suspended in 1 mL PBS and injected via the tail vein 6 times at a one-week interval. The rats in the DM+PBS group were infused with 1 mL PBS. One week after the last treatment of MSCs, all rats were sacrificed by cervical decapitation, and blood and lung samples were collected for further assessment.

### 2.4. Serum Biochemistry

The total triacylglycerol and total cholesterol were detected by the Department of Laboratory Medicine of West China Hospital, Sichuan University (Chengdu, China).

### 2.5. Measurement of MDA and GSH Activities

The activities of malondialdehyde (MDA) and micro reduced glutathione (GSH) in lung tissue were determined using an MDA Detection Kit (Solarbio, Beijing, China) and a Micro Reduced GSH Assay Kit (Solarbio, Beijing, China) according to the manufacturer's protocols.

### 2.6. Histopathology

For histological examination, rat lung tissue was fixed in 10% neutral-buffered formalin for 48 h, paraffin-embedded, and sectioned at an average thickness of 5 *μ*m. The sections were stained with hematoxylin and eosin (H&E), Masson, Periodic acid-Schiff (PAS), and Sirius Red.

### 2.7. Immunohistochemistry

Paraffin sections were dewaxed in xylene and rehydrated with decreasing concentration of ethanol, followed by antigen retrieval in 0.01 mol/L citrate buffer. Endogenous peroxidase was quenched with 3% H_2_O_2_, and unspecific binding sites were blocked with normal goat serum (Solarbio). The sections were incubated with primary antibodies against *α*-SMA, HMGB1, MMP9, P65, NLRP3, GPX-1, GST, Nrf2, and fibronectin overnight at 4°C. Then, the sections were incubated with a biotin-labeled secondary antibody followed by incubation with HRP-labeled streptavidin. Thereafter, the signals were detected with 3,30-diaminobenzidine, counterstained with hematoxylin, and observed under an optical microscope (Zeiss, Germany).

### 2.8. Apoptosis Assay

Apoptosis was assessed in paraffin-embedded lung sections with the use of a terminal deoxynucleotidyl transferase dUTP nick end labeling (TUNEL, Beyotime, China) in situ cell death detection kit according to the manufacturer's instructions.

### 2.9. Western Blot

The total protein samples from lung tissues were homogenized using RIPA lysis buffer containing protease inhibitors (Calbiochem, San Diego, USA) and phosphatase inhibitors (Calbiochem). The protein concentrations of the samples were determined using the BCA Protein Assay Kit (Beyotime). The protein samples were loaded onto the sodium dodecyl sulfate-polyacrylamide gel electrophoresis (SDS-PAGE) gel (10–12.5%), separated electrophoretically, and transferred onto polyvinylidene difluoride membranes (Millipore Corporation, Billerica, USA). After blocking with 5% nonfat milk for 1 h, the membrane was individually incubated overnight at 4°C with the primary antibodies listed in Table 1. Then, the membrane was incubated at room temperature for 1.5 h with horseradish peroxidase-conjugated antibodies at a 1 : 4000 dilution. The proteins were visualized by enhanced chemiluminescence (Amersham, UK) reagents in the Molecular Imager Gel Doc XR System (Bio-Rad, Hertfordshire, UK). Protein bands were quantified by NIH ImageJ software and normalized to *β*-actin.

### 2.10. Statistical Analysis

All statistical analyses were performed using GraphPad Prism v8.0 (GraphPad Prism, CA, USA) and expressed as the mean ± standard deviation (SD). All data were assessed for normality of distribution using the Kolmogorov-Smirnov and Shapiro-Wilk tests before data analysis, and differences between groups were analyzed using one-way ANOVA with Tukey's *post hoc* analysis or Kruskal-Wallis *H* with Student-Newman-Keuls (SNK) *post hoc* analysis. Statistical significance was defined as *P* ≤ 0.05.

## 3. Results

### 3.1. MSCs Inhibit Epithelial-Mesenchymal Transition and Fibrosis in Lung Tissue of Diabetic Rats

**Figure 1:**
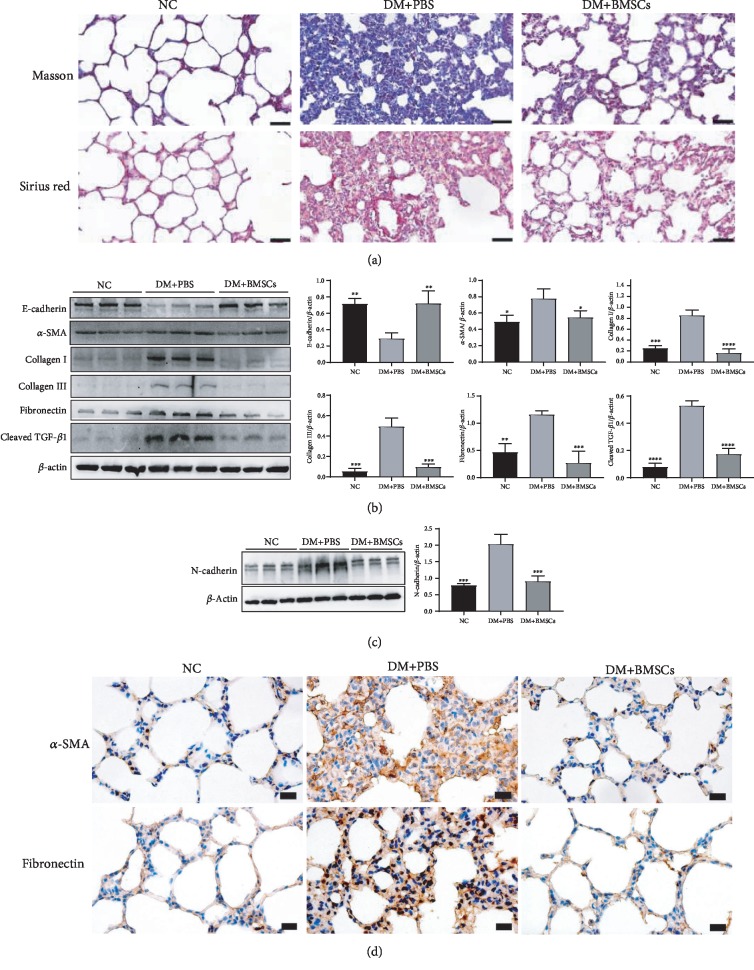
MSCs inhibit lung fibrosis caused by diabetes in rats. (a) Masson and Sirius Red staining of lung tissues. Magnification, ×400. Scale bar, 50 *μ*m. (b, c) Effects of MSCs on the protein expressions of E-cadherin, *α*-SMA, collagen I, collagen III, fibronectin, cleaved TGF-*β*1, and N-cadherin by the western blotting assay. (d) The representative micrographs of immunohistochemical staining of *α*-SMA and fibronectin. Magnification, ×200. Scale bar, 20 *μ*m. Data are shown as mean ± standard deviation (^∗^*P* < 0.05, ^∗∗^*P* < 0.01, ^∗∗∗^*P* < 0.001, and ^∗∗∗∗^*P* < 0.0001 compared with the DM+PBS group, *n* = 6 per group).

Lung tissue collagen content was analyzed by Masson staining and Sirius Red staining (Figure 1(a)). Collagen deposition obviously increased in the diabetic rat lung tissues compared with the control rat tissues, while it apparently decreased in the DM+BMSC group compared with the DM+PBS group.

Pulmonary fibrosis is characterized by the conversion of lung fibroblasts to myofibroblasts and excessive deposition of ECM proteins such as type I, III, IV, and VI collagen, resulting in reduced gas exchange and impaired lung function. Therefore, we examined the expression of epithelial-mesenchymal transition (EMT) and fibrosis-associated biomarkers in lung tissues. As shown in Figures 1(b) and 1(c), diabetic rat lung tissue showed significant increases in the levels of N-cadherin, *α*-SMA, collagen I, collagen III, fibronectin, and cleaved TGF-*β*1, with concomitant decreases in the levels of E-cadherin, compared to those in the control rat tissue; meanwhile, compared with the DM+PBS group, these changes were significantly inhibited or even reversed in the DM+BMSC group. In addition, the immunohistochemistry assay also showed that the expression levels of *α*-SMA and fibronectin were markedly decreased in the DM+BMSC group than in the DM+PBS group (Figure 1(d)).

### 3.2. MSCs Activate the Sirt3/SOD2 Signaling Pathway

**Figure 2:**
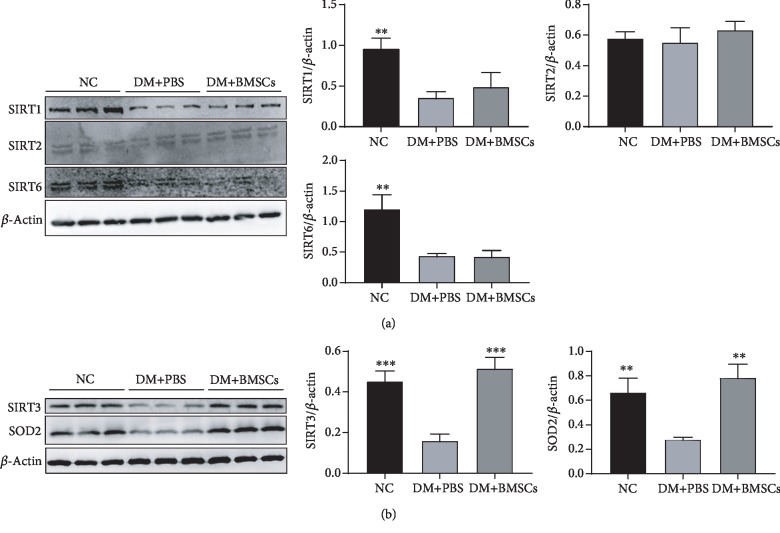
MSCs activate Sirt3 signal in diabetic rats. (a) Effects of MSCs on the protein expressions of Sirt1, Sirt2, and Sirt6 in the lung tissue by the western blotting assay. (b) Effects of MSCs on the protein expressions of Sirt3 and SOD2 in the lung tissue by the western blotting assay. Data are shown as mean ± standard deviation (^∗∗^*P* < 0.01, ^∗∗∗^*P* < 0.001 compared with the DM+PBS group, *n* = 6 per group).

It is well known that the sirtuin family is composed of seven members (Sirt1 to Sirt7), out of which Sirt1, Sirt2, Sirt3, and Sirt6 exert positive effects on tissue fibrosis, especially Sirt3 [41, 42]. To investigate the mechanisms of MSCs against diabetes-induced lung fibrosis, we hypothesized that the capability of MSCs might mainly result from upregulating the Sirt3/SOD2 signaling pathway. As shown in Figures 2(a) and 2(b), we have observed significant reduction in protein expressions of Sirt1, Sirt3, Sirt6, and SOD2 in diabetic rat lung tissue, except Sirt2, compared to those in the control rat tissue. Administration of MSCs results in a significant increase in Sirt3 and SOD2 protein expressions but fails to upregulate the protein expressions of Sirt1 and Sirt6, suggesting that Sirt3/SOD2 signaling pathway signaling may participate in the protective effect of MSCs against diabetic lung fibrosis.

### 3.3. MSCs Reduce Oxidative Stress via the Nrf2/ARE Signaling Pathway

**Figure 3:**
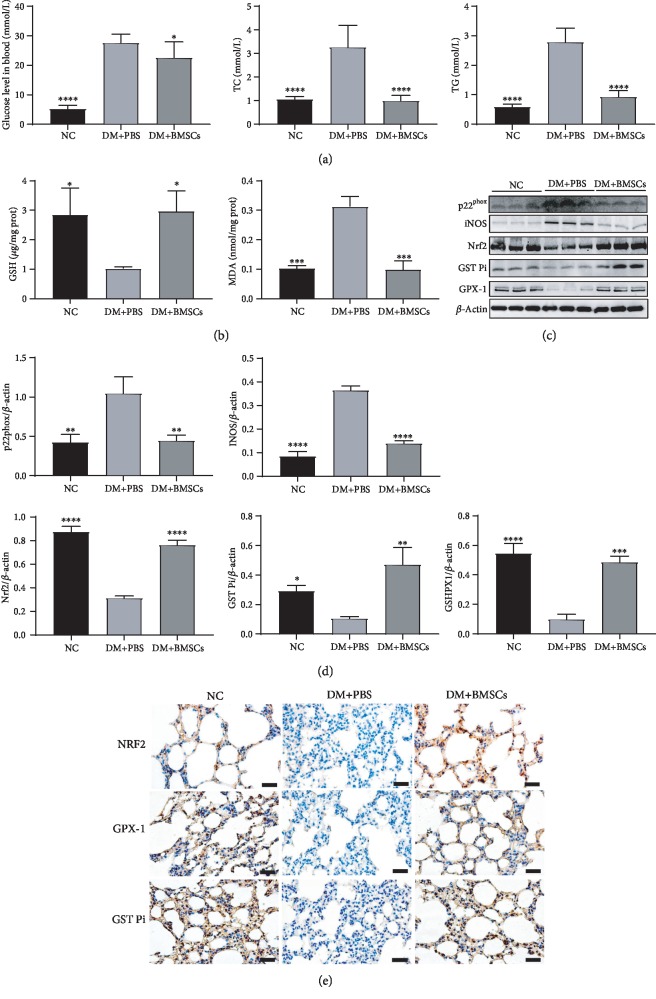
MSCs attenuate lung oxidative stress in diabetic rats. (a) Levels of blood glucose, total cholesterol, and triglyceride. (b) Effects of MSCs on the levels of GSH and MDA. (c) Effects of MSCs on the protein expressions of p22^phox^, iNOS, Nrf2, GST Pi, and GPX-1 in the lung tissue by the western blotting assay. (d) Quantitative analysis of the levels of p22^phox^, iNOS, Nrf2, GST Pi, and GPX-1. (e) The representative micrographs of immunohistochemical staining of Nrf2, GPX-1, and GST Pi. Magnification, ×200. Scale bar, 50 *μ*m. Data are shown as mean ± standard deviation (^∗^*P* < 0.05, ^∗∗^*P* < 0.01, ^∗∗∗^*P* < 0.001, and ^∗∗∗∗^*P* < 0.0001 compared with the DM+PBS group, *n* = 6 per group).

Diabetes is a chronic metabolic disease characterized by hyperglycemia, which is always accompanied by elevated blood triglyceride and cholesterol levels. Microenvironment with high fat and high glucose usually causes oxidative stress. Oxidative stress is implicated as an important molecular mechanism underlying fibrosis in a variety of organs, including the lungs. To assess the effects of MSCs on diabetes, changes in blood glucose, triglycerides, and total cholesterol were measured. As shown in Figure 3(a), the levels of triglyceride and total cholesterol were significantly higher in the DM+PBS group than in the control group, while they were significantly lower in the DM+BMSC group than in the DM+PBS group. However, the effect of MSCs on improving glycemia was not obvious.

Levels of MDA, GSH, iNOS, and NADPH oxidase subunit p22^phox^ in the lung tissues reflect the degree of oxidative stress. Higher levels of MDA, iNOS, NADPH oxidase subunit p22^phox^ and lower levels of GSH were found in the DM+PBS group as compared to the control group (Figures 3(b)–3(d)). Meanwhile, the levels of MDA, iNOS, and NADPH oxidase subunit p22^phox^ contents were significantly decreased, while the level of GSH was significantly increased in the DM+BMSC group compared with the DM+PBS group. These results demonstrate that MSCs effectively reduce oxidative stress.

NF-E2-related factor 2 (Nrf2) is a basic region leucine-zipper transcription factor that binds to the antioxidant response element (ARE) and thereby regulates the expression of a large battery of genes involved in the cellular antioxidant and anti-inflammatory defense. Immunohistochemistry and western blot analyses showed that the protein levels of Nrf2, glutathione-S-transferase pi (GST Pi), and glutathione peroxidase 1(GPX-1) significantly increased in the DM+MSC group compared with the DM+PBS group (Figures 3(c)–3(e)), suggesting that MSCs may reduce lung oxidative stress by activating the Nrf2/ARE signal pathway.

### 3.4. MSCs Attenuate Inflammation via Inhibiting the NF-*κ*B/HMGB1/RAGE/NLRP3 Signaling Pathway

**Figure 4:**
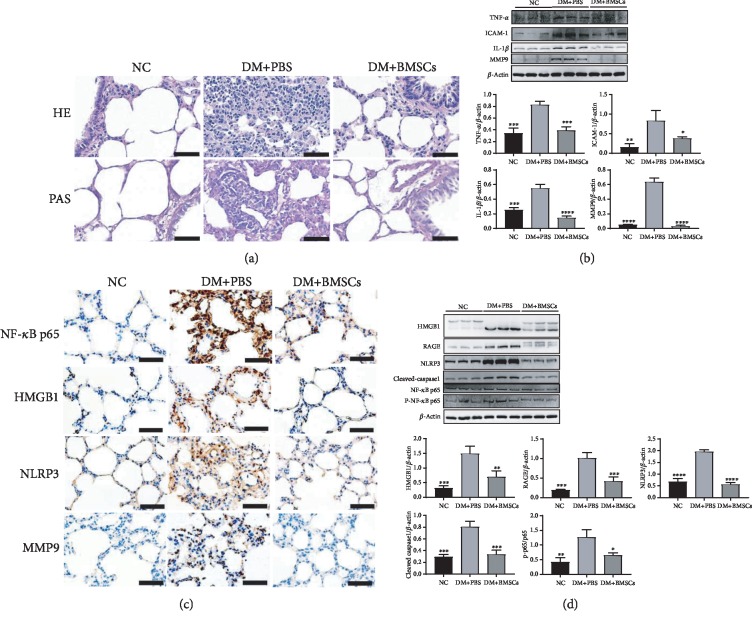
MSCs attenuate lung inflammation in diabetic rats. (a) The representative micrographs of HE and PAS staining of the lung tissue. Magnification, ×400. Scale bar, 50 *μ*m. (b) Effects of MSCs on the protein expressions of TNF-*α*, ICAM-1, IL-1*β*, and MMP9 in the lung tissue. (c) The representative micrographs of immunohistochemical staining of p65, HMGB1, NLRP3, and MMP9. Magnification, ×200. Scale bar, 50 *μ*m. (d) Effects of MSCs on the protein expressions of HMGB1, RAGE, NLRP3, cleaved caspase-1, p65, and p-p65. Data are shown as mean ± standard deviation (^∗^*P* < 0.05, ^∗∗^*P* < 0.01, ^∗∗∗^*P* < 0.001, and ^∗∗∗∗^*P* < 0.0001 compared with the DM+PBS group, *n* = 6 per group).

HE and PAS staining of lung tissues was performed to evaluate lung injury. Rats in the control and the DM+BMSC groups showed normal lung structures with thin alveolar walls and light PAS staining (Figure 4(a)). In contrast, lung tissues of rats in the DM+PBS group showed thickening alveolar walls, increased inflammatory cell infiltration, and darker PAS staining.

As shown in Figure 4(b), the expression levels of TNF-*α*, ICAM-1, IL-1*β*, and MMP9 were also significantly lower in the DM+BMSC group than in the DM+PBS group. Furthermore, the cytoplasmic translocation of HMGB1, the nuclear translocation of NF-*κ*B p65, and the NF-*κ*B/HMGB1/RAGE/NLRP3/caspase-1 signaling pathway were significantly inhibited by MSCs compared with the DM+PBS group (Figures 4(c) and 4(d)).

### 3.5. MSCs Protect Lung Tissue Cells from Apoptosis via Activating the AKT Pathway

**Figure 5:**
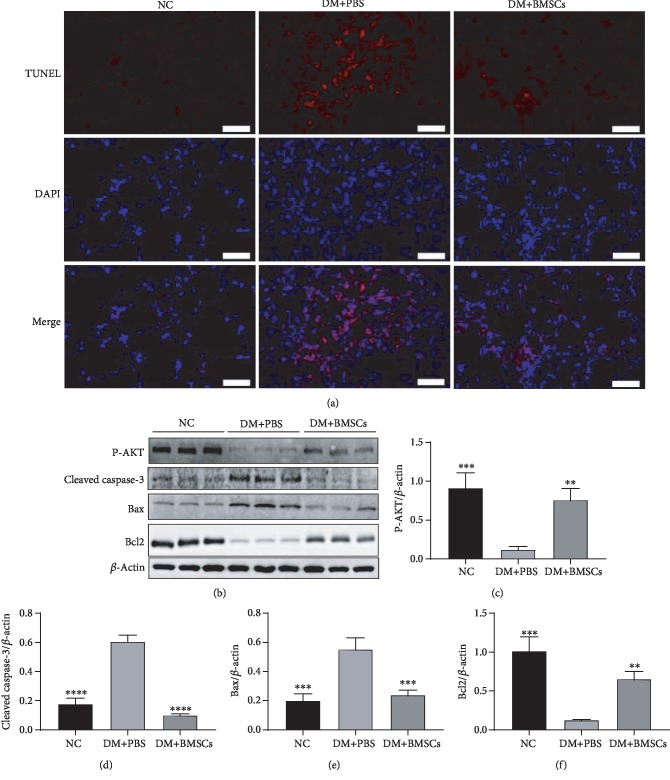
MSCs protect lung tissue cells from apoptos is in diabetic rats. (a) The representative micrographs of TUNEL staining of lung sections. Magnification, ×200. Scale bar, 50 *μ*m. (b) Effects of MSCs on the protein expressions of P-AKT, cleaved caspase-3, Bax, and Bcl2 in the lung tissue by the western blotting assay. (c–f) Quantitative analysis of the levels of P-AKT, cleaved caspase-3, Bax, and Bcl2. Data are shown as mean ± standard deviation (^∗^*P* < 0.05, ^∗∗^*P* < 0.01, ^∗∗∗^*P* < 0.001, and ^∗∗∗∗^*P* < 0.0001 compared with the DM+PBS group, *n* = 6 per group).

TUNEL staining showed that diabetes induced a significant increase in apoptosis in the lung section of the diabetic rats compared with the control rats (Figure 5(a)), while MSC treatment partially reversed these changes. We further analyzed Bcl-2, Bax, cleaved caspase-3, and p-AKT expression by western blotting, and the results showed that MSC treatment significantly upregulated the levels of Bcl-2 and p-AKT in lung tissues, while it significantly decreased the levels of Bax and cleaved caspase-3 (Figures 5(b)–5(f)). These results reveal that MSCs may protect lung tissue cells from apoptosis via activating the AKT pathway.

### 3.6. MSCs Enhance Autophagy of Lung Tissue in Diabetic Rats

**Figure 6:**
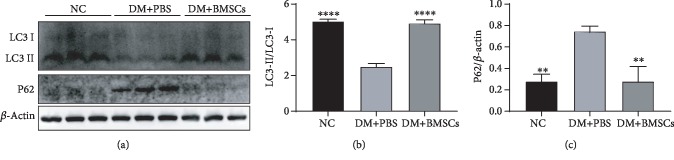
MSCs enhance autophagy in the lung of diabetic rats. (a) Effects of MSCs on the protein expressions of LC3 and P62 in the lung tissue by the western blotting assay. (b, c) Quantitative analysis of the levels of LC3 and p62. Data are shown as mean ± standard deviation (^∗∗^*P* < 0.01, ^∗∗∗∗^*P* < 0.0001 compared with the DM+PBS group, *n* = 6 per group).

To elucidate the effect of MSCs on autophagy in the lungs of diabetic rats, we examined the expression of autophagic signaling markers using western blotting analyses. Remarkably, we found increased expression levels of microtubule-associated protein LC3-II/LC3-I and decreased level of p62 in the DM+BMSC group compared with the DM+PBS group (Figures 6(a)–6(c)).

### 3.7. MSCs Inhibit Endoplasmic Reticulum Stress of Lung Tissue in Diabetic Rats

**Figure 7:**
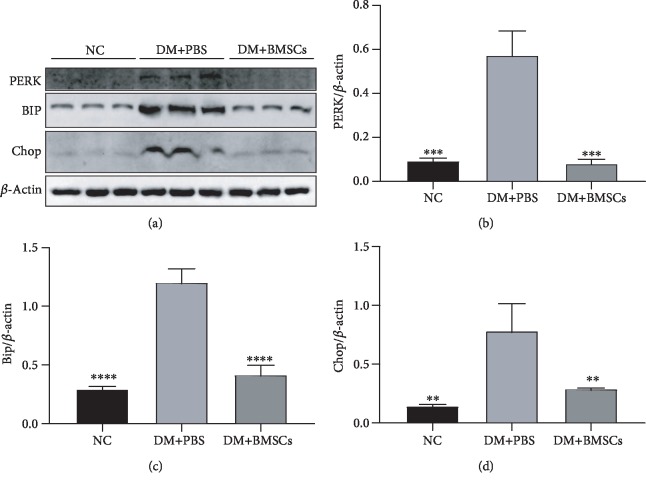
MSCs attenuate endoplasmic reticulum stress in the lung of diabetic rats. (a) Effects of MSCs on the protein expressions of PERK, BIP, and Chop in the lung tissue by the western blotting assay. (b–d) Quantitative analysis of the levels of PERK, BIP, and Chop. (^∗∗^*P* < 0.01, ^∗∗∗^*P* < 0.001, and ^∗∗∗∗^*P* < 0.0001 compared with the DM+PBS group, *n* = 6 per group).

Bip, Chop, and PERK are ER stress markers regulated by ER function. The expression levels of them measured by the western blot were significantly higher in the DM+PBS group than in the control group, while they were significantly lower in the DM+MSC group than in the DM+PBS group (Figures 7(a)–7(d)).

## 4. Discussion

In recent years, lung injury caused by diabetes has received increasing attention, and it has been reported that the lung could be one of the target organs of diabetes based on clinical findings [10, 43–45]. However, there was not enough evidence at the animal level to elucidate the pathogenesis of diabetic lung injury. Although MSCs are found to ameliorate a variety of diabetic complications, the effect of MSCs against diabetic lung injury is unclear. In this study, we tried to explore whether MSCs have protective effects against diabetic lung injury and found that MSCs could attenuate diabetic lung fibrosis by adjusting Sirt3-mediated oxidative stress, inflammation, apoptosis, autophagy, and endoplasmic reticulum stress, not only providing insights into the biochemical mechanisms of lung injury in diabetes but also establishing theoretical foundation for further exploration of MSC-based diabetic lung fibrosis therapeutics.

Many previous studies have demonstrated that sustained hyperglycemia caused fibrotic changes in multiple organs, such as the kidney, heart, skin, and liver. A few other studies have also used a diabetic rat model to study the impact of hyperglycemia on fibrotic changes in the lung [11, 46, 47]. Lung fibrosis is caused by abnormal proliferation of myofibroblasts and fibroblasts, which secrete excessive ECM proteins including fibronectin, laminin, and collagens [48]. Epithelial-mesenchymal transition (EMT) is a process by which polarized immotile epithelial cells convert to motile mesenchymal cells. EMT is characterized by the loss of proteins associated with the polarized epithelial phenotype such as E-cadherin and an increase in mesenchymal markers such as N-cadherin and *α*-SMA [49]. Although the exact origin of activated myofibroblasts remains uncertain, recent studies showed that the EMT process is essential in the pathogenesis of lung fibrosis [50, 51]. In the present work, EMT and lung fibrosis were observed in diabetic rats, and this could be reversed, at least in part, by MSCs via enhancing autophagy and suppressing inflammation, oxidative stress, apoptosis, and ER stress, suggesting that MSCs play protective effects against diabetic lung fibrosis. There is increasing evidence that Sirt3 plays an important role in the process of lung fibrosis [24, 29], and it is closely related to inflammation, oxidative stress, apoptosis, autophagy, and ER stress [31, 52–54]. Based on western blotting assays, we found that the protein level of Sirt3 in lung tissue was significantly decreased in diabetic rats, while it can be markedly upregulated by MSCs, suggesting that Sirt3-mediated signaling may participate in the protective effect of MSCs against diabetic lung fibrosis.

Oxidative stress is believed to be critical in the pathogenesis of lung fibrosis [55–58]. Microenvironment with high fat and high glucose can induce the production of large amounts of reactive oxygen species in the energy metabolic system, which may further harm multiple tissues and organs throughout the body. To evaluate the effects of MSCs on diabetes, the levels of blood glucose, triglyceride, and total cholesterol were measured. Compared with the normal rats, diabetic rats showed higher blood glucose, triglyceride, and total cholesterol levels, and MSC treatment could significantly reduce the glucose, triglyceride, and total cholesterol levels in diabetic rats. Assessment of the levels of MDA, GSH, iNOS, and NADPH oxidase subunit p22^phox^ revealed a high level of oxidative stress in the lung tissue of diabetic rats. It has been reported that activation of SOD2 by Sirt3 is beneficial for the removal of superoxide in the regulation of oxidative stress [59, 60]. Furthermore, activation of Sirt3 is known to promote the Nrf2/ARE pathway [61]. Overexpression of Sirt3 reduced the level of SOD2 acetylation and stimulated Nrf2 translocation to regulate oxidative stress [62]. In the present study, we found that MSCs could enhance the antioxidative capacity via upregulating the Sirt3 level, promoting the protein expressions of SOD2, Nrf2, GST Pi, and GPX-1, and suppressing the levels of MDA, GSH, iNOS, NADPH oxidase subunit p22^phox^, suggesting that MSCs can attenuate oxidative injury via adjusting Sirt3 signal and against diabetic lung fibrosis.

Lung fibrosis is characterized by inflammatory and fibroproliferative changes, including the release of inflammatory cytokines such as interleukins (IL-1*β*, IL-4, and IL-13), TNF-*α*, IFN-*γ*, matrix metalloproteinases (MMPs) and transforming growth factor *β* (TGF-*β*), and transcription factors such as nuclear factor-*κ*B (NF-*κ*B) [63]. These inflammatory cytokines lead to epithelial cell damage, fibroblastic proliferation, and excessive deposition of ECM proteins, resulting in the destruction of the alveolar structure [48]. High-mobility group 1 (HMGB1) is a nonhistone nuclear protein that can promote inflammation when released extracellularly after cellular activation, stress, damage, or death [64]. Extracellular HMGB1 plays a central role in mediating injury and inflammation, and interactions involving HMGB1-TLR- (Toll-like receptors-) RAGE (receptor for advanced glycation endproducts) constitute a tripod that triggers NF-*κ*B activation [65]. The NOD-like receptor protein 3 (NLRP3) inflammasome is composed of NLRP3, the apoptosis-associated speck-like protein containing a caspase recruitment domain (ASC) and caspase-1, and it controls caspase-1 activation that regulates the maturation of IL-1*β* and IL-18. Recent reports have shown that NLRP3 and ASC/caspase-1/IL-1*β* signaling are important for HMGB1 induction and release, although the exact mechanism is unclear [66–68]. Another study shows that HMGB1 induces NLRP3 activation via NF-*κ*B [69]. Recent studies suggested that reduction of the Sirt3 expression induced the activation of HMGB1, NF-*κ*B, NLRP3, and caspase-1 and upregulation of the Sirt3 expression inhibited the activation of NF-*κ*B, HMGB1, NLRP3, and caspase-1 [52, 70, 71]. In the present study, our results revealed that MSCs effectively reduced the expression levels of p-NF-*κ*B p65/NF-*κ*B p65, HMGB1, RAGE, NLPRP3, and cleaved caspase-1 and then significantly inhibited the levels of IL-1*β*, TNF-*α*, ICAM-1, and MMP9. These data indicate that MSCs increase Sirt3 expression and then inhibit the activation of the NF-*κ*B/HMGB1/NLRP3 pathway to improve inflammatory response to diabetic lung fibrosis.

Apoptosis plays an important role in the pathophysiology of DM [72, 73] and also is involved in the pathogenesis of lung fibrosis [63]. Recently, hyperglycemia-induced apoptosis has been extensively studied on the balance of the proapoptotic protein Bax and the antiapoptotic protein Bcl2 toward apoptosis. The expression of caspase-3, a key apoptosis executioner, can induce apoptosis. It has been reported that inhibiting the phosphorylation of Akt and FoxO family proteins is related to cell apoptosis [74]. Sirt3 was found to exert its antiapoptotic effect by regulating the Akt signaling pathway [54]. Thus, a high level of Sirt3 can adjust the Akt signaling pathway to suppress apoptosis. In our studies, MSC treatment significantly decreased Bax and cleaved caspase-3 expression and increased Bcl-2 expression in the lung via increasing the expression levels of p-AKT. Both cell proliferation and cell death can modulate alveolar repair, and we observed a decrease in apoptosis in the lungs of diabetic rats after MSC treatment. These data suggested that the protective effect of MSCs against diabetic lung fibrosis may be through inhibiting apoptosis via upregulating the Sirt3 signal.

Autophagy is a molecular mechanism that maintains cellular physiology and promotes survival. Defects in autophagy lead to the causes of many diseases, such as diabetes mellitus, cancer, neurodegeneration, and infection disease, and to aging [75]. Recently, insufficient autophagy has been believed to be one of the important features in the pathogenesis of lung fibrosis [76, 77]. However, few papers outline the autophagic alteration in diabetic lung injury. Both LC3 and p62 are frequently used as biomarkers to assess autophagy [78]. In recent studies, Sirt3 is known to regulate autophagy via the deacetylation of several autophagy-related genes (ATGs), which play important roles in autophagy [53]. In this study, we found that MSCs significantly enhanced autophagy reducing the expression levels of p62 and increasing the ratio of LC3-II/LC3-I. Taken together, MSCs upregulated the Sirt3 level to enhance autophagy, thereby attenuating diabetic lung fibrosis.

Endoplasmic reticulum (ER) stress is associated with the development and progression of fibrotic diseases, including lung fibrosis [18]. The glucose-regulated protein GRP78 is a 78 kDa protein called immunoglobulin heavy chain binding protein (BiP/GRP78), which is the major molecular chaperone in the endoplasmic reticulum [79]. PKR-like endoplasmic reticulum kinase (PERK) is believed to be sensors of ER stress [80]. It has been reported that PERK induces apoptosis by the accumulation of CCAAT/enhancer-binding protein homologous protein (CHOP) under irremediable ER stress [81]. Recent studies showed that Sirt3 was an integral regulator of ER function and that its depletion might result in the hyperacetylation of critical ER proteins that protected against islet lipotoxicity under conditions of nutrient excess [31]. In our studies, MSC treatment significantly decreased BIP, CHOP, and PERK expression compared to the DM+PBS group. These data suggested that MSC treatment significantly ameliorated ER stress by adjusting the Sirt3 signal against diabetic lung fibrosis.

In addition, the role of endogenous lung mesenchymal stem cells (lung MSCs) in lung injury repair has received increasing attention in recent years [82]. An interesting study recently showed that a population of Dermo1^+^ mesenchymal cells (endogenous MSCs) served as a reservoir for epithelial cell regeneration and reestablishment of the normal airway epithelium during mouse lung LPS and naphthalene injury repair [83]. Although MSCs could repair various tissue injuries, few papers have demonstrated whether intravenously transplanting MSCs could induce the activation of lung MSCs to attenuate lung injury. Therefore, further understanding of the role of lung MSCs in diabetic lung fibrosis may provide invaluable insights into MSC-based diabetic therapeutics.

Moreover, there are also several limitations to this study. First, in the present study, we have observed significant reduction in protein expressions of Sirt1, Sirt3, and Sirt6 in the DM+PBS group, except Sirt2, compared with the control rats. This observation might support the hypothesis that Sirt1, Sirt3, and Sirt6 could play an important role in the development of tissue fibrosis induced by diabetes, which is consistent with previous research results [24, 41, 42] and Sirt2 might not participate in this process. Administration of MSCs results in a significant increase in Sirt3 protein expressions but fails to upregulate the protein expressions of Sirt1 and Sirt6, suggesting that the protective effect of MSCs against diabetic lung fibrosis may be sirt3-dependent, which may be related to the paracrine effects of MSCs [84], but the specific mechanism is not clear. Since there are few reports to elucidate the effects and mechanisms of MSCs on sirtuins, future studies on the current topic are therefore recommended. In addition, although existing research reports show that the expression level of the sirtuin protein is consistent with the change in activity level [85–87], the activity of sirtuins in diabetic lung tissue needed to be detected for further study. Second, we did not investigate the specific mechanisms of MSCs on diabetic lung fibrosis that is composed of nearly 40 different cell types in vitro. Third, we next intend to use STZ-induced diabetes in Sirt3-deficient mice as a model and investigate whether the protective effects of MSCs on lung injury are Sirt3-dependent. Finally, we mainly focused on the role of Sirt3-mediated stress responses which involve multiple pathways in diabetic lung fibrosis; the specific mechanisms of each particular pathway are not enough and needed to be further investigated.

## 5. Conclusions

In conclusion, our study demonstrates that MSCs effectively ameliorate diabetic lung fibrosis via regulating Sirt3-mediated stress responses, including enhancement of autophagy and inhibition of oxidative stress, inflammation, apoptosis, and endoplasmic reticulum stress. These results not only shed new light on the pathogenesis of diabetic lung injury but also provide a theoretical foundation for further exploration of diabetic lung fibrosis therapeutics.

## Figures and Tables

**Table 1:** The information of the antibodies used in the present work.

Antibody	Source	Dilutions	Company
E-cadherin	Mouse	1 : 1000	BD Biosciences, San Jose, USA
*α*-SMA	Rabbit	1 : 500	Wanleibio, Shenyang, China
Collagen I	Rabbit	1 : 500	Wanleibio, Shenyang, China
Collagen III	Rabbit	1 : 300	Wanleibio, Shenyang, China
Fibronectin	Rabbit	1 : 500	Wanleibio, Shenyang, China
N-cadherin	Rabbit	1 : 2000	Huabio, Hangzhou, China
SIRT1	Rabbit	1 : 500	Huabio, Hangzhou, China
SIRT2	Rabbit	1 : 1000	Huabio, Hangzhou, China
SIRT3	Rabbit	1 : 500	Wanleibio, Shenyang, China
SIRT6	Rabbit	1 : 2000	Abcam, Cambridge, UK
SOD2	Rabbit	1 : 1000	Wanleibio, Shenyang, China
p22^phox^	Rabbit	1 : 500	Wanleibio, Shenyang, China
iNOS	Rabbit	1 : 500	Wanleibio, Shenyang, China
Nrf2	Rabbit	1 : 1000	Wanleibio, Shenyang, China
GST Pi	Rabbit	1 : 500	Wanleibio, Shenyang, China
GSHPX1	Rabbit	1 : 1000	Wanleibio, Shenyang, China
TNF-*α*	Rabbit	1 : 500	Wanleibio, Shenyang, China
ICAM-1	Rabbit	1 : 500	Wanleibio, Shenyang, China
IL-1*β*	Rabbit	1 : 500	Wanleibio, Shenyang, China
Cleaved TGF-*β*1	Rabbit	1 : 1000	Wanleibio, Shenyang, China
MMP9	Rabbit	1 : 1000	Wanleibio, Shenyang, China
HMGB1	Rabbit	1 : 2500	Huabio, Hangzhou, China
RAGE	Rabbit	1 : 1000	Bimake, Shanghai, China
NLRP3	Rabbit	1 : 1500	Wanleibio, Shenyang, China
Cleaved caspase-1	Rabbit	1 : 500	Wanleibio, Shenyang, China
NF-*κ*B p65	Rabbit	1 : 500	Wanleibio, Shenyang, China
P-NF-*κ*B p65	Rabbit	1 : 1000	CST, Boston, USA
P-AKT	Rabbit	1 : 500	Wanleibio, Shenyang, China
Cleaved caspase-3	Rabbit	1 : 500	Wanleibio, Shenyang, China
Bax	Rabbit	1 : 500	Wanleibio, Shenyang, China
Bcl2	Rabbit	1 : 500	Wanleibio, Shenyang, China
LC3	Rabbit	1 : 1000	CST, Boston, USA
p62	Rabbit	1 : 500	Wanleibio, Shenyang, China
PERK	Rabbit	1 : 1000	Wanleibio, Shenyang, China
BIP	Rabbit	1 : 1500	Wanleibio, Shenyang, China
Chop	Rabbit	1 : 500	Wanleibio, Shenyang, China
*β*-Actin	Rabbit	1 : 5000	ABclonal, Woburn, USA

## Data Availability

All data included in this study are available upon request by contact with the corresponding author.
